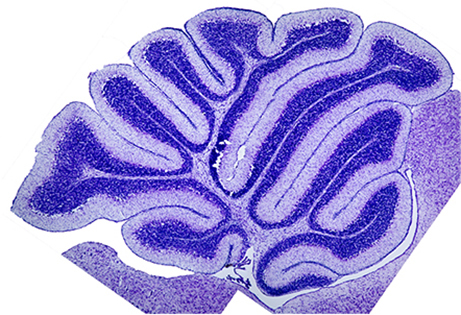# A mouse model for neonatal jaundice

**Published:** 2014-09

Neonatal jaundice, which is caused by high serum levels of unconjugated bilirubin, is usually a temporary condition caused by delayed induction of UGT1A1, the enzyme that conjugates bilirubin for excretion. However, uncontrolled hyperbilirubinemia can cause permanent neurological damage. Here, Muro et al. study the mechanism of bilirubin neurotoxicity *in vivo* by generating mice that bear a null mutation in the *Ugt1* gene. By investigating bilirubin neurotoxicity in two genetic backgrounds, the researchers show that susceptibility to bilirubin damage is strain-specific, thereby underscoring the importance of modifier genes in the modulation of bilirubin toxicity. In addition, by exposing the mice to phototherapy, which converts unconjugated bilirubin to a water-soluble form, at different postnatal times, they identify a window of neuronal susceptibility to bilirubin toxicity. These findings establish *Ugt1*-null mice as a versatile model in which to study bilirubin neurotoxicity and potential treatments for hyperbilirubinemia. **Page 1057**

**Figure:**